# MD-Miner: a network-based approach for personalized drug repositioning

**DOI:** 10.1186/s12918-017-0462-9

**Published:** 2017-10-03

**Authors:** Haoyang Wu, Elise Miller, Denethi Wijegunawardana, Kelly Regan, Philip R.O. Payne, Fuhai Li

**Affiliations:** 1 Department of BioMedical Informatics (BMI), The Ohio State University, Columbus, OH 43210 USA; 2 College of Engineering, The Ohio State University, Columbus, OH 43210 USA; 3 College of Engineering, Northeastern University, Boston, MA 02115 USA; 4 Colledge of Art and Science, The Ohio State University, Columbus, OH 43210 USA; 5 Institute for Informatics, Washington University in St. Louis School of Medicine, St. Louis, MO 63110 USA

**Keywords:** Network, Personalized medicine, Precision medicine, Drug repositioning, Mechanism of action

## Abstract

**Background:**

Due to advances in next generation sequencing technologies and corresponding reductions in cost, it is now attainable to investigate genome-wide gene expression and variants at a patient-level, so as to better understand and anticipate heterogeneous responses to therapy. Consequently, it is feasible to inform personalized drug treatment decisions using personal genomics data. However, these efforts are limited due to a lack of reliable computational approaches for predicting effective drugs for individual patients. The reverse gene set enrichment analysis (i.e., connectivity mapping) approach and its variants have been widely and successfully used for drug prediction. However, the performance of these methods is limited by undefined mechanism of action (MoA) of drugs and reliance on cohorts of patients rather than personalized predictions for individual patients.

**Results:**

In this study, we have developed and evaluated a computational approach, known as Mechanism and Drug Miner (MD-Miner), using a network-based computational approach to predict effective drugs and reveal potential drug mechanisms of action at the level of signaling pathways. Specifically, the patient-specific signaling network is constructed by integrating known disease associated genes with patient-derived gene expression profiles. In parallel, a drug mechanism of action network is constructed by integrating drug targets and z-score profiles of drug-induced gene expression (pre vs. post-drug treatment). Potentially effective candidate drugs are prioritized according to the number of common genes between the patient-specific dysfunctional signaling network and drug MoA network. We evaluated the MD-Miner method on the PC-3 prostate cancer cell line, and showed that it significantly improved the success rate of discovering effective drugs compared with the random selection, and could provide insight into potential mechanisms of action.

**Conclusions:**

This work provides a signaling network-based drug repositioning approach. Compared with the reverse gene signature based drug repositioning approaches, the proposed method can provide clues of mechanism of action in terms of signaling transduction networks.

## Background

The average cost of developing a new drug is about 2.6 billion dollars, as reported in a study conducted by Tuft’s Center for the Study of Drug Development [1]. The estimated success rate of drugs in clinical trials for FDA approval is ~12%, a key contributor to huge development costs [1]. With ~2000 currently FDA-approved small molecule drugs [2, 3], roughly over 15,000 compounds that are well studied and passed toxicity tests [4, 5] had entered into clinical trials but eventually failed. Due to advances in next generation sequencing (NGS) technologies and corresponding reductions in cost [6], it is now possible to investigate genome-wide gene expression and variants at the individual patient-level, so as to better understand and anticipate heterogeneous responses to therapy. Systematic genomics analyses have revealed diversity of dysfunctional biomarkers of cancer samples [7–10], which is believed to be responsible for heterogeneous drug responses of individual patients [11].

By integrating patients’ personal genomics data, e.g., genome wide gene expression and encoding structural variation profiles, and publicly available pharmacogenomics big data [9, 10, 12], it is possible to reposition FDA approved drugs and agents tested in clinical trials for new indications, in a fast and cheap manner, to yield effective personalized anticancer therapies [5, 13–16]. For example, commercial companies are developing data-driven computational approaches and software tools for personalized drug predictions, such as Foundation Medicine [17], as well as the Verge Genomics platform for brain disorders [18]. The widely used data resources and tools being used for this type of research are the Connectivity Map and LINCS [12] projects, which successfully provide the open-source data (z-score profiles of drugs) and tools for applications of drug sensitivity prediction [19], drug repositioning [20–23], and drug combination therapy [24, 25]. However, the mechanism of action of predicted drugs often remain unknown. Elucidating drugs’ mechanism of action is an important challenge in pharmacology requiring the specific molecular targets of given drugs, as well as the consequent actions (signaling transductions pathways) originating from drug targets. Further, such understanding is of significant importance when seeking to translate these types of findings into early-stage validation and clinical studies. To overcome such challenges, in this study, we propose a computational approach, mechanism and drug miner (MD-Miner), for drug repositioning, using a network-based approach. The mechanism of action signaling network of drugs and disease signaling network of individual patients are constructed via said methodology by integrating protein-protein interactome data with gene expression data of individual patients and drugs, and then predicting effective drugs for individual patients based on the constructed signaling networks.

## Results

### Method overview

**Fig. 1:**
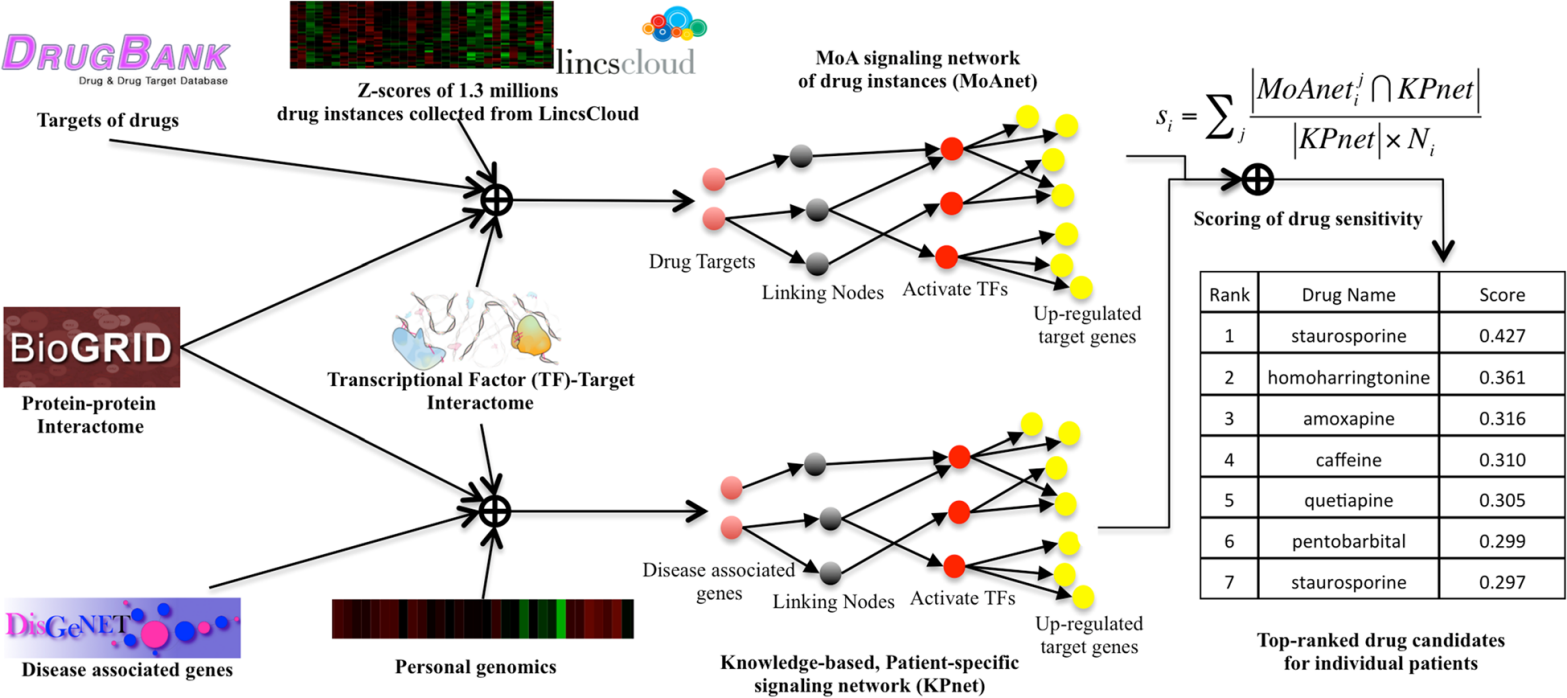
Method Overview. There are three modules: Module 1): Construction of mechanism of action (MoA) signaling network (MoAnet) of drug instances (the same drug treatment on different cell lines with different doses and time); Module 2): Construction of patient-specific disease signaling network (Pnet); and Module 3): Scoring of drug sensitivity. For each drug, the average network overlapping nodes between MoAnet and Pnet are calculated and used as the drug sensitivity score for individual patients, and then drugs are ranked based on the sensitivity score in the decreasing order. The top-ranked drugs have higher possibility to be effectively repositioned for given individual patients

Figure 1 shows the overview of the drug prediction method consisting of three major modules. Module 1): Construction of mechanism of action (MoA) signaling network (MoAnet) of drug instances, comprised of 1.3 million drug and genetic perturbation instances derived from different cell lines, drug doses and data collection times, as found in CMap/LINCS [12]. Target information for said drugs is obtained using the DrugBank database [2, 3]. Subsequently, activated transcription factors (TFs) are identified based on up-regulation of TF target genes integrating TF-target interactome data [26], and the z-score profiles of drug instances generated by Connectivity Map [12] (available via LincsCloud [27]). Finally, drug targets, activated TFs and their up-regulated target genes are mapped onto the BioGRID [28] protein-protein network (interactome) in order to construct the “MoAnet” using Dijkstra’s algorithm [29]. Module 2): Construction of patient-specific disease signaling networks (Pnet). The same method used in MoAnet construction is employed to link disease associated genes (knowledge) obtained from DisGeNET [30, 31], activated TFs and up-regulated target genes based on personal genomics data of individual patients (patient-specific). Module 3): Scoring of drug sensitivity. For each drug, the average network overlapping nodes between MoAnet and Pnet are calculated and used as the drug sensitivity score for individual patients, and then drugs are ranked based on the sensitivity score in the decreasing order.

### Drug repositioning for prostate cancer using PC-3 cell line

Prostate cancer is the second most common type of cancer, where 1 in 7 men in U.S. will be diagnosed with prostate cancer. Prostate cancer is also the second-leading cause of cancer-related death in American men [32]. Due to the widespread incidence and leading cancer-related death rate, a significant proportion of clinical studies are related to prostate cancer treatment. In this study, we evaluate the proposed approach using the PC-3 prostate cancer cell line as a use case, and will improve and apply the proposed method on different type of cancers and diseases in our future work.

### Pnet construction for PC-3 cell line

**Fig. 2:**
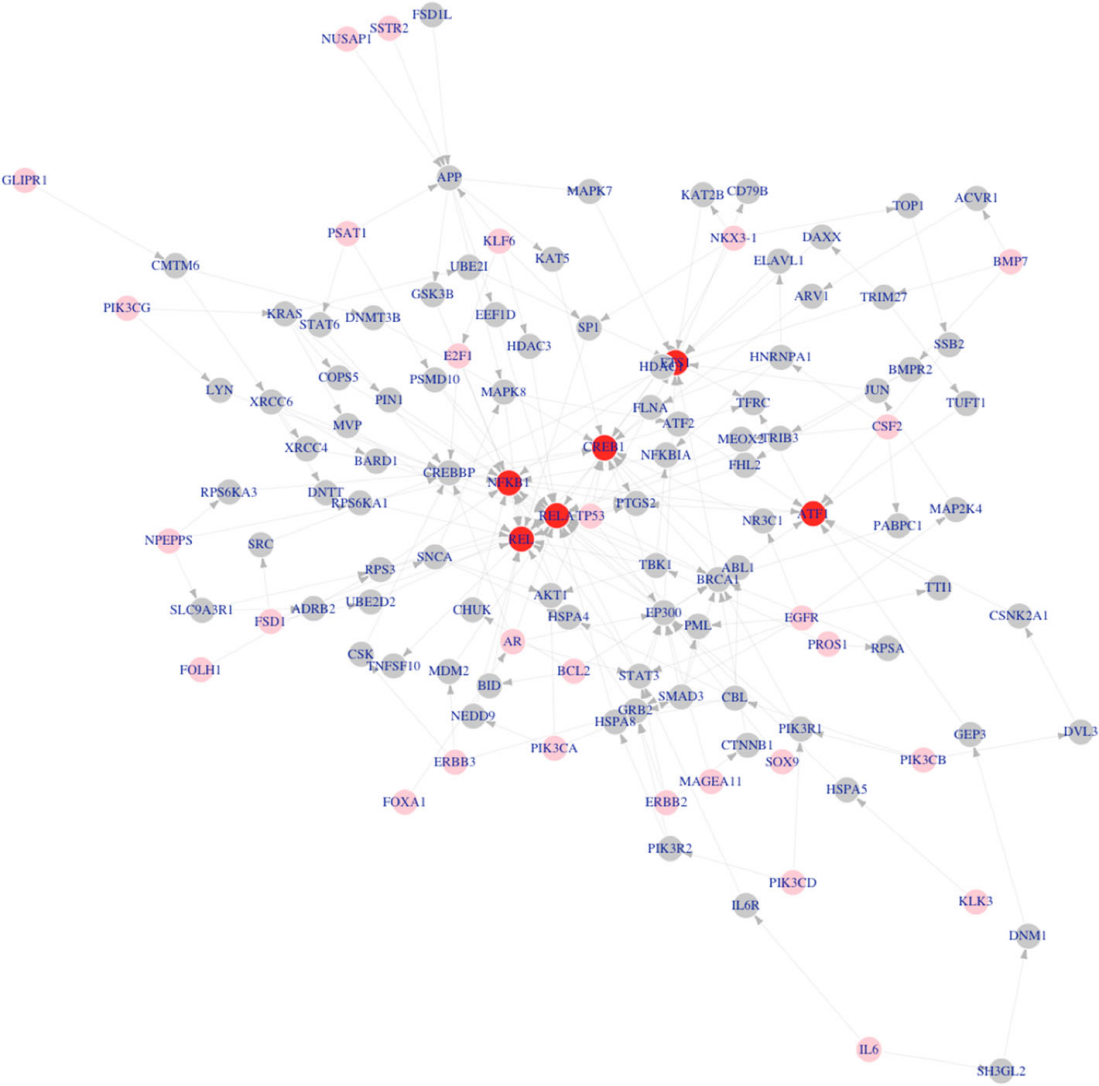
Sub-network of reconstructed patient signaling network (*Pnet*) of PC-3 cell line (prostate cancer). There are 121 genes (nodes) and 214 interactions (edges). *Pink*, *gray* and *red* color represents disease-associated genes, linking genes and activated transcriptional factors

Gene expression data of PC-3 (prostate cancer) and RWPE-1 (normal prostate) cell lines were generated by V. Härmä et al., in [33] (available at GEO: GSE19426). The average gene expression of duplicates of PC-3 and RWPE-1 are used to calculate the fold change of gene expression. From DisGeNET, the top 30 prostate cancer associated genes are collected, which are listed in Table 1. Twenty-four transcriptional factors, as shown in Table 2, are identified as activated (with the threshold *T = 2)* in PC-3 cell line. There are eight up-regulated (fold change > = 2) target genes of the 24 activated TFs. All the disease-associated genes, activated TFs and up-regulated target genes are mapped onto the BioGRID protein-protein interaction network, the Pnet of PC-3 is constructed by linking the disease associated genes (source nodes) with activated TFs (target nodes) together, and then linking the TFs with their target genes, in which 237 genes (nodes) and 647 interactions (edges) are included. Figure 2 shows part of the constructed Pnet of PC-3 cell line, in which 121 genes (nodes) and 214 interactions (edges) are included. Pink, gray and red colors represent disease-associated genes, linking genes and activated transcriptional factors.

**Table 1:** Top 30 prostate cancer associated genes obtained from DisGeNET

BCL2	EGFR	PIK3CA	PIK3CB	FSD1L
AR	ERBB2	IL6	PROS1	PSAT1
SOX9	ERBB3	SSTR2	PIK3CG	NPEPPS
TP53	E2F1	PIK3CD	NKX3-1	FOLH1
MAGEA11	FOXA1	CSF2	FSD1	GLIPR1
KLF6	BMP7	KLK3	NUSAP1	PLAG1

**Table 2:** Twenty-four activated TFs in PC-3 cell line

ATF2	PPARG	JUN	USF1
NFKB1	HIF1A	CEBPB	NFATC1
RELA	RXRB	PPARD	RARB
ETS1	ATF1	CREB1	NFATC4
REL	NFKB2	NFATC2	NFATC3
RXRA	TFAP2A	RXRG	NFAT5

### MoAnet construction of FDA approved drugs

**Fig. 3:**
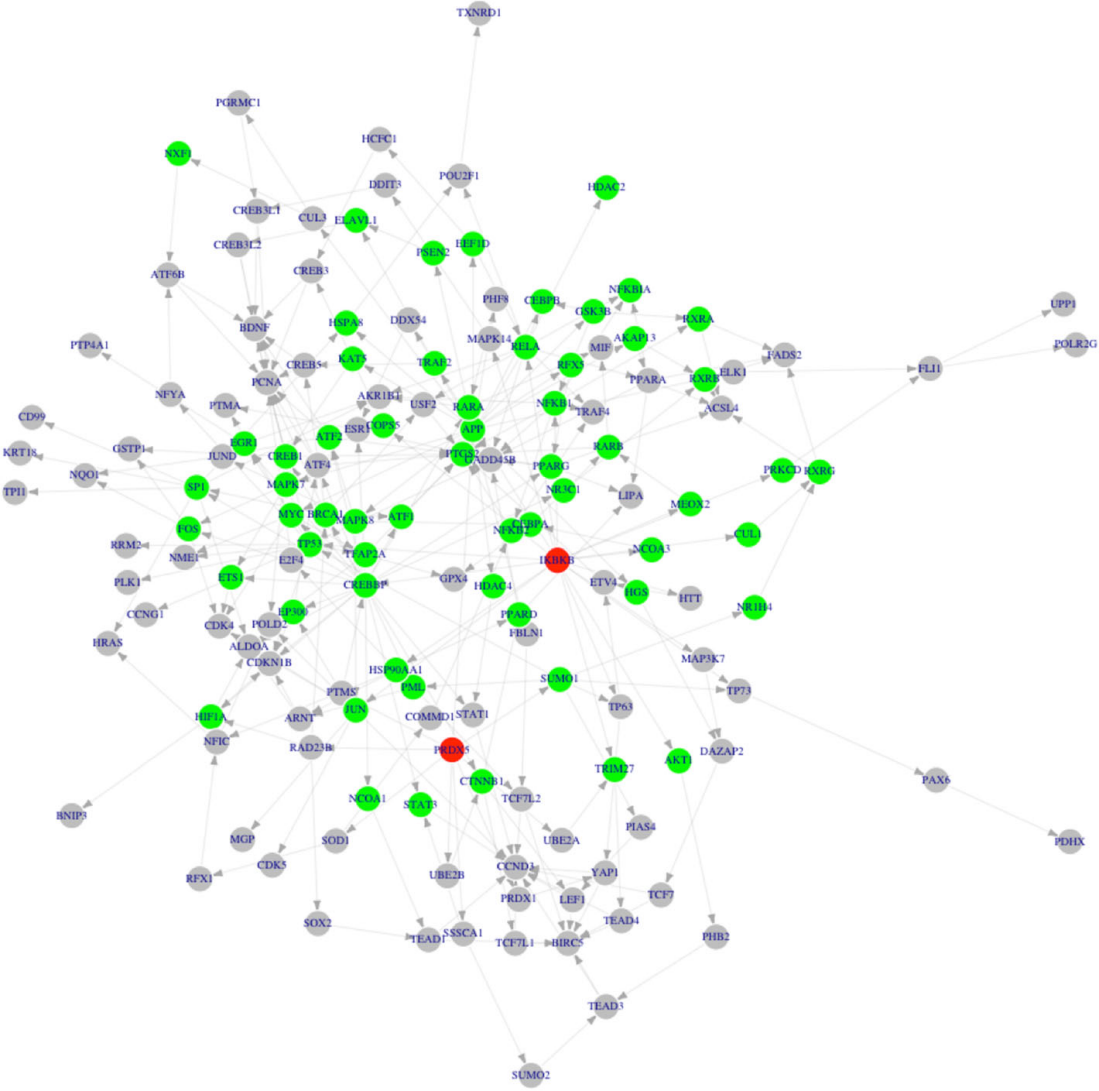
MoAnet of Auranofil instance on A549 cell line. There are 121 genes (nodes) and 214 interactions (edges). *Red, gray* and *green* color represents drug targets, linking genes and common genes appeared in both Pnet of PC-3 and MoAnet of Auranofil instance

The DrugBank database [2, 3] is the most widely used database for querying drug information, e.g., drug targets and mechanism, that currently contains 8206 drug entries, including 2202 U.S. Food and Drug Administration (FDA) approved drugs (1991 FDA-approved small molecule drugs, 211 FDA-approved biotech (protein/peptide) drugs), and over 6000 experimental drugs. The target information obtained from DrugBank includes 11,957 drug-target interactions between 4797 drugs and 2245 targets (6510 drug-target interactions between 1456 FDA approved drugs and 1447 targets). The z-score data (genomics data) of 1.3 million of drug instances were obtained from Connectivity Map [12] via LincsCloud [27]. In total, 1160 drugs, including 1058 FDA approved agents, and their 32,053 z-score profiles (treated on different cell lines with 24 h and 10 uM dose) were obtained. Consequently, the MoA signaling network of 36,107 (including 32,053 FDA approved drug instances) were calculated using the same method of Pnet construction using drug target information and z-score profiles of drug instances. Figure 3 shows an example MoAnet of Auranofin (CMAP ID: BRD-A79465854, CMAP Instance ID: HOG003_A549_24H_X3_F1B10/G03) (Prediction rank: 7, Score of sensitivity: 0.255, Growth inhibition rate on PC-3 cell line: −63.994) on A549 (lung cancer) cell line. The green nodes indicate the network overlap between Pnet of PC-3 and MoAnet of Auranofil instance on A549 cell line. As can be seen, there are a large number of overlapping network nodes, which indicates the potential effectiveness of auranofil on PC-3 cell line.

### Drug repositioning and evaluation

**Fig. 4:**
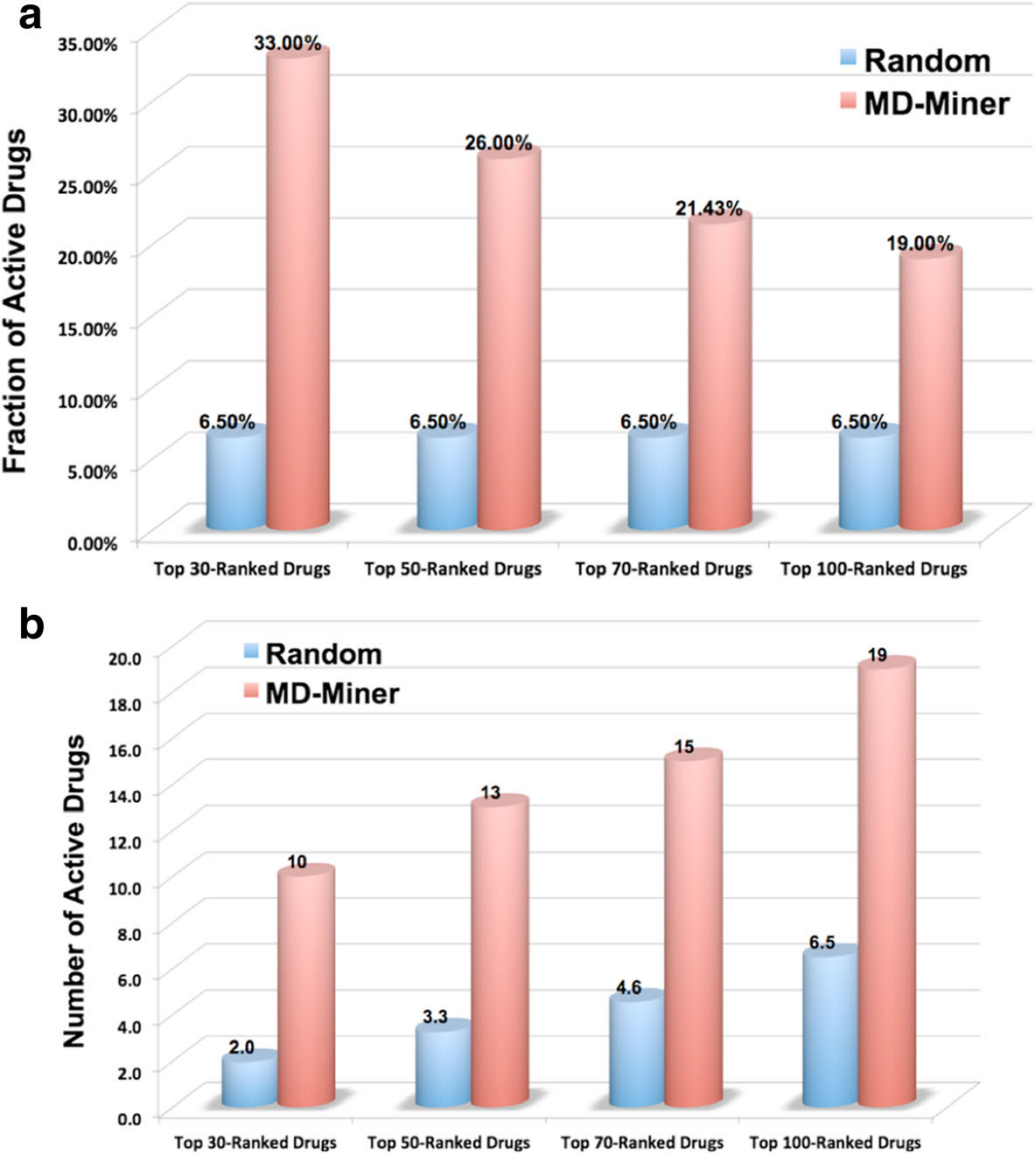
Evaluation of MD-Miner drug prediction results. The fraction and number of active drugs among the top-30, top-50, top-70, top-100 ranked drugs predicted by MD-Miner are shown in (**a**) and (**b**) respectively

In a recent drug screening study [34], 1398 drugs were evaluated on the PC-3 cell line, where the growth inhibition rate of drugs were made available online [34]. In total, 68 drugs were considered as potentially efficacious, as they reduced the mean growth rate to less than or equal to 1.5 standard deviations below the average across all agents (growth rate ≤ 54.57) [34]. Among the 1398 screened drugs, MoAnets were constructed that included 402 drugs that are contained in CMap/LINCS, including 394 FDA-approved drugs, along with target information and z-score profiles. Of the 402 selected drugs, 26 of the 68 active drugs were recovered in the constructed MoAnets. These drug numbers are summarized in Table 3. Drug sensitivity scoring for the PC-3 cell line was performed in order to rank the 402 drugs. Figure 4 shows the evaluation results (fraction of active drugs and number of active drugs among the top 30, 50, 70, 100 predicted results) of the prediction compared with random selection. As can be seen, the MD-Miner can improve the possibility of successful drug repositioning significantly (33.3% success rate in MD-Miner versus 6.5% in random selection) compared with random selection (the expectation values of the random selection are used here, rather than randomly select effective drugs repeatedly) (Fig. 4a). In another word, 10 out of 26 active drugs are identified among the top 30 predicted drugs (only 2 active drugs can be identified in random selection) (Fig. 4b). Table 4 shows the 10 active drugs among the top-30 prediction results. In addition to the well-known anti-cancer drugs, e.g., Docetaxel and Paclitaxel, the Auranofin (for inflammatory arthritis treatment) and Digoxin (for heart disease treatment) can inhibit tumor growth significantly.

**Table 3:** Number of drugs in different resources

# of drugs in ref. [35]	# of drugs in ref. [35] and CMAP	# of FDA approved drugs in ref. [35] and CMAP	# of potential efficacious drugs in ref. [35]	# of FDA approved, potential efficacious drugs in ref. [35] and CMAP
1398	402	394	68	26

**Table 4:** Ten active drugs in top-30 ranked drugs predicted by MD-Miner

Rank	Drug Name	Score of Sensitivity (Predicted)	Growth Inhibition Rate on PC3
1	Staurosporine	0.468	−16.375
4	Docetaxel	0.265	18.695
6	Paclitaxel	0.257	1.426
7	Auranofin	0.255	−63.994
12	Bortezomib	0.245	−74.068
13	Cladribine	0.244	2.411
15	Dactinomycin	0.236	−15.905
19	Homoharringtonine	0.226	−42.758
21	Digoxin	0.205	−71.924
30	Etoposide	0.188	43.669

## Discussion

There are still a few *limitations* of the proposed method that should be noted, including: 1) the use of in vitro assays or animal models derived from cancer patient samples is needed to prove the reliability of the proposed approach; 2) the measurement of genetic mutation data of individual patients is not currently integrated in the method. Patient-specific mutations, rather than general disease associated genes, can be integrated with patient specific gene expression in order to obtain accurate patient-specific signaling network; and 3) the construction of the MoAnet of drugs depends on the availability of known drug targets and z-score profiles from CMAP/LINCS. However, as shown in this study, the target information and z-score profiles of many drugs may not be available. Specifically, instead of using shortest path approach, gene expression fold-change information and sophisticated network construction approaches, e.g., a weighted network or clustered network analysis [35], should be evaluated to construct accurate MoAnet and Pnet signaling network. Finally, the reverse gene signature based drug prediction score should be combined with the network-based score to improve the drug prediction results. In the future work, we will improve the proposed method by solving these limitations, and will also apply and evaluate the proposed method on different type of cancers and diseases.

## Conclusion

Diverse and unique genomic variation in individual patients is believed to be responsible for heterogeneous drug response [9, 10]. Due to the advances made in NGS technology, it has become affordable for individual patients to be genotyped, resulting in the identification of clinically relevant and/or actionable genome-wide genetic variants. However, computational methods are needed to systematically integrate personal genomics data and other sources of big “omics” data characterizing drug potential efficacy in order to advance precision medicine for individual patients. Despite a few existing computational approaches that have been developed for drug prediction and repositioning [5, 12–16, 20–23], it remains an open questions as to how to integrate diverse data resources and predict effective drugs for individual patients. In contrast to traditional connectivity mapping approaches using differentially expressed genes, we have proposed a methodology to reposition drugs based on the mechanism of action signaling network of drugs and disease signaling network of individual patients that are constructed by integrating protein-protein interactome data with gene expression profiles of drugs and individual patients. The evaluation on the PC-3 prostate cancer cell line showed that it significantly improved the success rate of discovering effective drugs compared with the random selection, and could provide insight into potential mechanisms of action.

## Methods

### Genomics data of PC-3

Gene expression data of PC-3 (prostate cancer) and RWPE-1 (normal prostate) cell lines were generated by V. Härmä et al., in [33] (available at GEO: GSE19426).

### Drug screening data on PC-3

The mean growth rates across at least three separate experiments for each of the 1398 agents on PC-3 prostate cancer cell line is available in the supplementary materials of reference [34].

### Prostate cancer associated genes

Top-thirty (30) prostate associated genes obtained by using DisGeNET [30, 31] online database (data set was downloaded in June 2016).

### Genomics (z-score) profiles of drugs

From lincsCloud, 1,328,098 z-score profiles were downloaded via Amason S3 using Firefox’s S3Fox plugin (http://download.lincscloud.org/) (data set was download in May, 2016).

### Drug-target interaction

The target information obtained from DrugBank (released on 2016-04-20, version 4.5.0) includes 11,957 drug-target interactions between 4797 drugs and 2245 targets (6510 drug-target interactions between FDA approved 1456 drugs and 1447 targets).

### Transcriptional Factor (TF)-Target interaction data

The TF-target interaction data was obtained from Transcriptional Regulatory Element Database (TRED) [36], and KEGG signaling pathways [37]. In total, 2618 TF-target interactions, between 192 TFs and 649 target genes, were collected [26]. The processed data set was used and is available in the code of reference [26].

### Identification of activated transcriptional factors (TFs)

The average fold change of three target genes with greatest fold change (for TFs with three or more target genes), or average fold change of all target genes (for TFs with two or less target genes) was used to indicate their activation score. The TFs with activation score greater or equal to 2.0 (average fold change of target genes) are selected as activated TFs.

### BioGRID protein-protein interactome

BioGRID (version 3.4.140) [28], a widely used protein-protein database, was downloaded at http://thebiogrid.org/download.php. The self-interaction edges were removed.

### MoAnet and Pnet network construction

Source nodes (drug targets or disease associated genes), activated TFs and their up-regulated target genes are mapped onto the BioGRID protein-protein network. Then signaling network (MoAnet of drug instances and Pnet of PC-3 cell line) are constructed by linking source nodes, activated TFs and target genes using Dijkstra’s algorithm. In another word, the Dijkstra’s algorithm was used to find the shortest paths between each of the drug targets, or disease associated genes to each of the activated TFs.

### Score of sensitivity

Potential effective drugs are repositioned (prioritized) in the decreasing order of average common genes between Pnet and MoAnet of drug instances as follows:

$$ {s}_i={\sum}_j\frac{\left|{MoAnet}_i^j\kern1em KPnet\right|}{\left| KPnet\right|\times {N}_i} $$

where *S*_*i*_ is the score of sensitivity of the *i*-th drug, *MoAnet*_*i*_^*j*^ is the MoAnet of the *j*-th instance of the *i*-th drug, *N*_*i*_ denotes the number of instance of the *i*-th drug, and the |.| operator represents the number of elements in a set. MoAnet of drugs on PC-3 were removed for drug scoring.

## Abbreviations

CMAP: Connectivity map
FDA: Food and Drug Administration
LINCS: Library of integrated network-based cellular signatures
MoA: Mechanism of action
MoAnet: Mechanism of action signaling network
NGS: Next generation sequencing
Pnet: Patient-specific disease signaling network
TF: Transcriptional factor
